# Supplementary figures and images for: Remodeling of RNA-binding proteome and RNA-mediated regulation as a new layer of control of sporulation
Source: mSystems. 2025 Aug 15;10(9):e00496-25. doi: 10.1128/msystems.00496-25 (PMC12456014; doi:10.1128/msystems.00496-25)

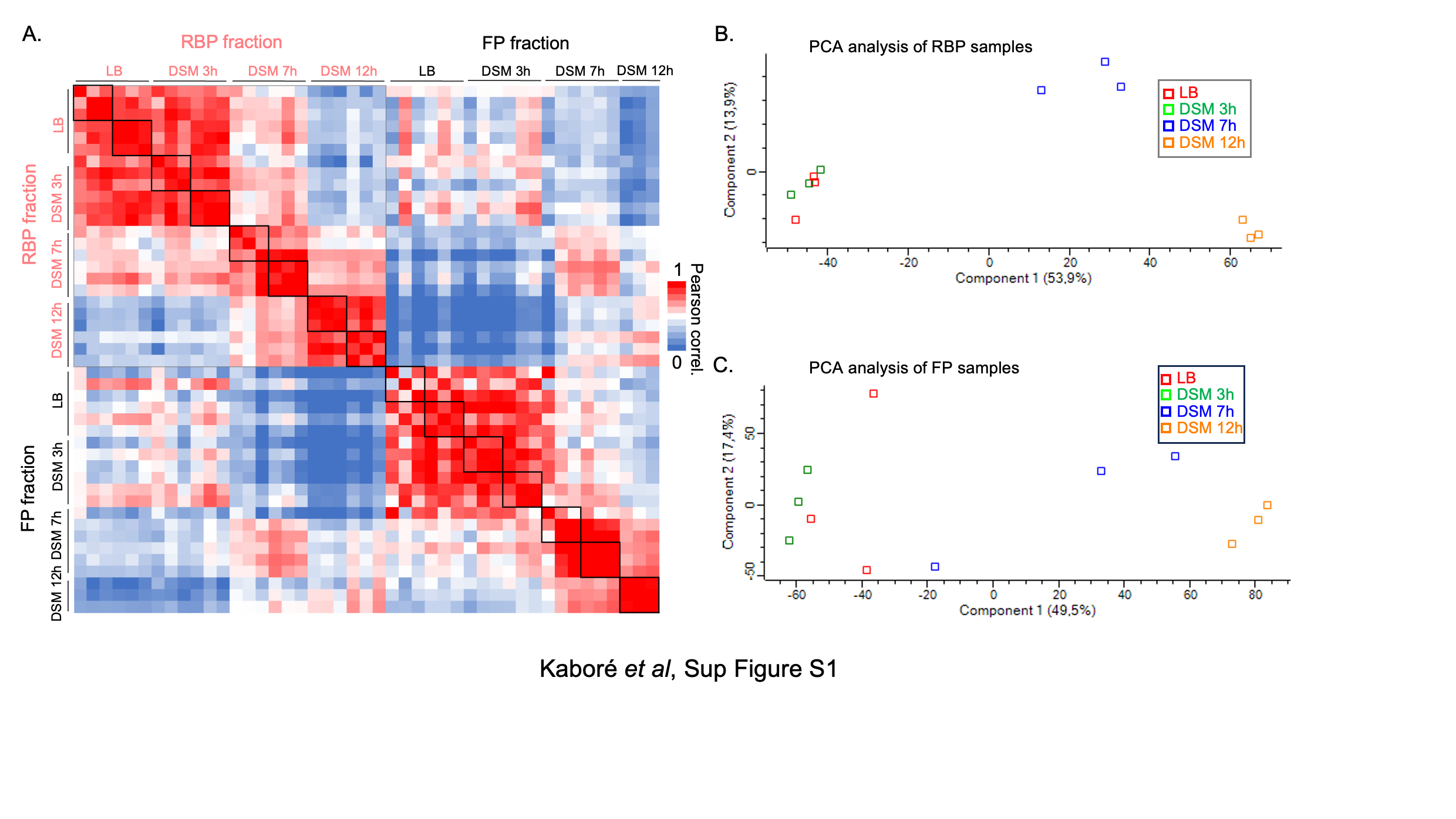

Supplement: Fig. S1 — Correlation matrix and principal component analysis. [file msystems.00496-25-s0001.tiff]

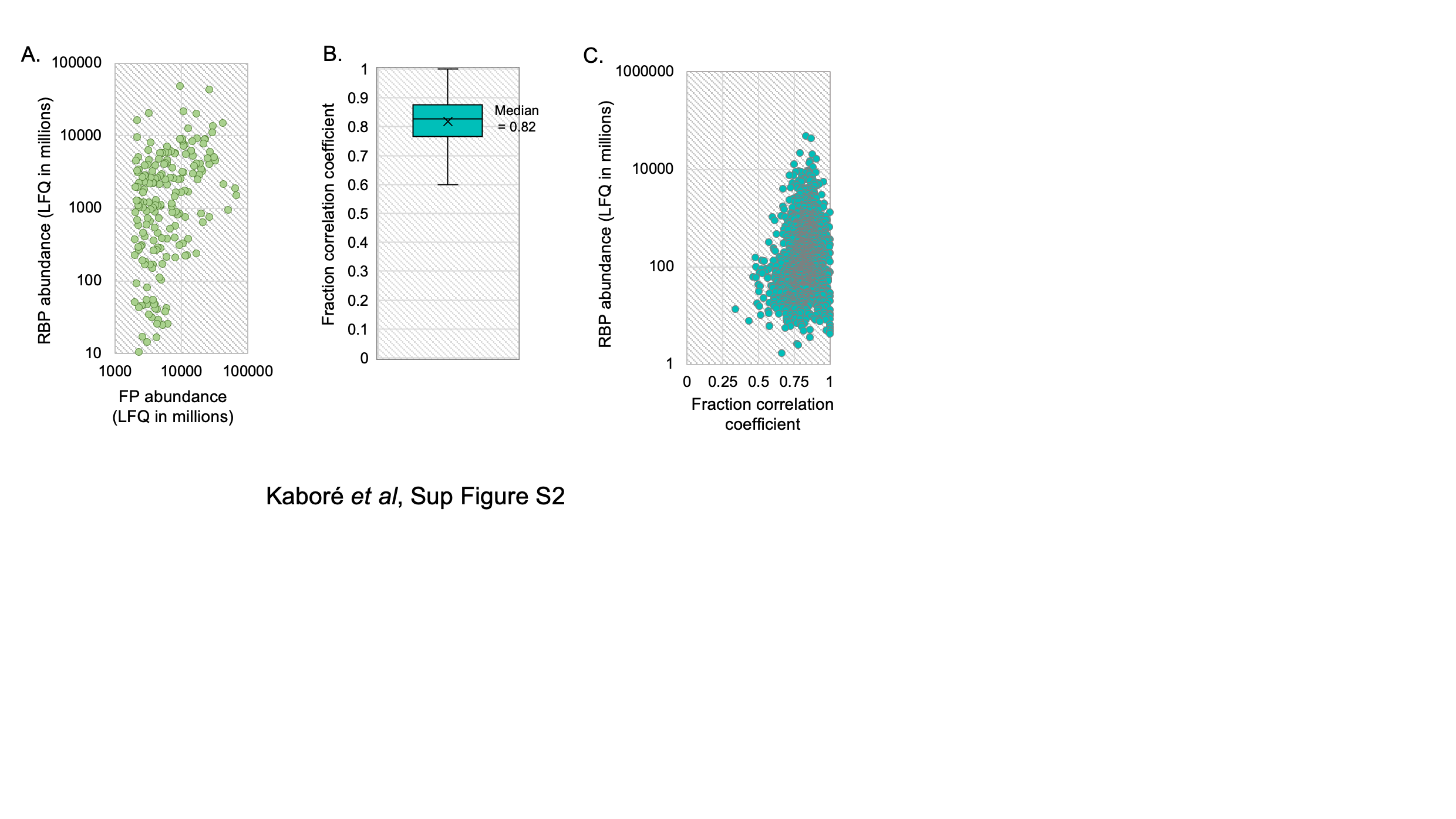

Supplement: Fig. S2 — LFQ intensities. [file msystems.00496-25-s0002.tiff]

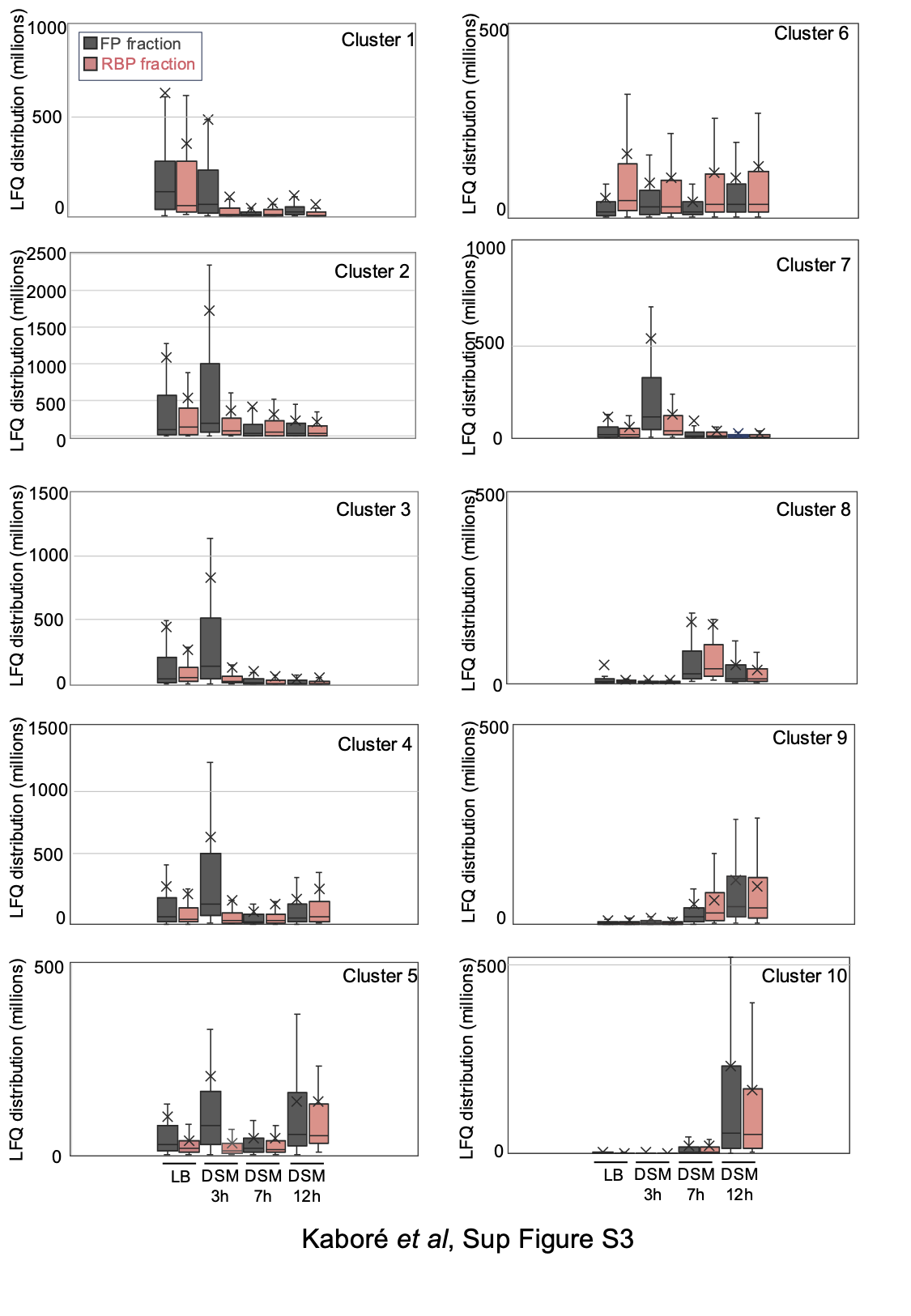

Supplement: Fig. S3 — Distributions of LFQ intensities of proteins within each cluster. [file msystems.00496-25-s0003.tiff]
